# Effects of Core Stability Training on Functional Movement Patterns in Tennis Players

**DOI:** 10.3390/ijerph192316033

**Published:** 2022-11-30

**Authors:** Joanna Majewska, Gabriela Kołodziej-Lackorzyńska, Barbara Cyran-Grzebyk, Daniel Szymczyk, Krzysztof Kołodziej, Piotr Wądołkowski

**Affiliations:** 1 Department of Physiotherapy, Institute of Health Sciences, College of Medical Sciences, University of Rzeszów, Aleja Majora Wacława Kopisto 2A, 35-315 Rzeszów, Poland; 2 Gawłowski Tennis Academy, ul. Piaseczyńska 71, 00-765 Warszawa, Poland

**Keywords:** functional movement screen test, core stability, tennis, screening, injury prediction, sport injuries, injury risk

## Abstract

The aim of this study was to assess the effects of a six-week core stability training program on the fundamental movement pattern assessed using the Functional Movement Screen Test in tennis players. The study group consisted of 160 subjects (74 women, 86 men) with a mean age of 20.26 ± 1.55. The Functional Movement Screen Test (FMS™), as well as the core stability tests including the lateral trunk muscles endurance test (the side bridge test), the abdominal muscles endurance test and the trunk extensors muscle endurance test—were used to evaluate the effects of core stability training. Statistical analysis revealed significant differences in the FMS test scores before and after introducing a core stability exercise program. Initially, the average total score of the FMS test in female tennis players was 14.58 ± 2.91, and after core stability training it was 17.20 ± 1.68 (*p* < 0.001). In the male group, the total FMS test score was 14.44 ± 2.76 before and 16.91 ± 1.36 after (*p* < 0.001) in the final assessment. Additionally, statistically significant differences were observed in the core stability test scores before and after introducing a stabilisation training program. The results of the study showed that specific core strengthening exercises could improve the FMS test scores in adult tennis players. This may also have an influence on reducing injury risk in this group, although further studies would be required to test this.

## 1. Introduction

Tennis is the world’s second most popular sport. It is played in 195 countries and it has an estimated group of 87 million fans around the world, which represents 1.17% of the world’s population [1,2]. This sport has an intermittent character with repetitive high-intensity effort (i.e., acceleration, deceleration, changes of direction, and strokes) over a variable period of competition time (i.e., on average 90 min). It demands a high level of physical fitness concerning speed, agility, muscle strength and power, as well as cardiovascular fitness to achieve high performance level [3]. Over the last few years, competitiveness in tennis has increased significantly, and players have devoted a significant time to improving their tennis skills through strength and conditioning, technical and tactical training, with an average of 15–20 h of training per week, even at a young age [4]. 

Due to the high demands placed on the body during training and competition, tennis players are at increased risk of many injuries, including chronic overuse syndromes and acute traumatic injuries [5]. Data from one of the most recent epidemiological studies on tennis injuries showed that upper limb injuries account for 28% of all injuries for male, adult players and 23% for female athletes, while the shoulder joint was reported to be the most frequently injured site of the upper limb [6]. The most common overuse tennis-related injuries include: internal impingement and superior labrum anterior-to-posterior (SLAP) tears in the shoulder, medial or lateral elbow tendinopathy, tendinitis and subluxation of the extensor carpi ulnaris tendon at the wrist, as well as abdominal muscle strains, lumbar muscle strains and lumbar disc degenerative pathologies. The lower extremities are more prone to acute injuries such as ankle sprains, meniscal knee injuries, knee joint tendinopathy and hip injuries [5]. 

There is still only limited evidence that total body and lower-extremity warm-up programs have the potential to significantly enhance performance and prevent injuries [7,8]. Correct fundamental movement patterns during sports and physical activities are very important for both athletic performance and injury prevention. The assessment of dysfunctional movement patterns can help healthcare professionals and coaches in implementing appropriate rehabilitation programs after injury, prescribing corrective exercise as well as developing injury-prevention training programs. 

The Functional Movement Screen test (FMS™) has been described as a screening tool used to assess the quality of basic movement patterns and side-to-side limitation and movement asymmetries [9]. The FMS was not intended to be a clinical, diagnostic tool. It was developed to evaluate movement performance in seven fundamental movement patterns in subjects with no current pain complaint or musculoskeletal injury. The seven tests in the FMS are: deep squat, hurdle step, in-line lunge, shoulder mobility, active straight-leg raise, trunk stability push-up, and rotary stability [10]. These movement patterns are scored from 0 to 3 (with 3 points being the optimal score), based on individuals‘ performance with or without pain or compensation. The scores of each test are then summed up for a maximal composite score of 21. Low composite scores in this test (14 points or lower), as well as side-to-side functional asymmetries are reported as increased injury risk factors in male and female athletes and male military candidates [11,12,13]. 

Previous studies have also evaluated the effects of different intervention programs focused on coordination, neuromuscular control, and/or strength training involved in FMS scores as a reliable measurement of movement skills and physical performance [14,15,16]. However, these studies considered sporting disciplines other than tennis. To the best of the authors‘ knowledge, there is an absence of such studies considering the impact of core strengthening exercise programs on FMS results in adult tennis players. Thus the aim of this study was to evaluate whether the implementation of the six-week core stability training program could improve FMS test scores in adult male and female tennis players.

## 2. Materials and Methods

### 2.1. Study Participants 

The study group included 160 adult tennis players from Polish tennis clubs (10 clubs from Warsaw and 4 clubs from Rzeszow) with an age range of 18 to 23 (mean age = 20.27 ± 1.68 years). The study group consisted of 74 women (mean age = 20.29 ± 1.83 years) and 86 men (mean age = 20.26 ± 1.55 years). The shortest training experience for all subjects ranged from 1 to 3 years (47.50% of the study group), while 52.50% of athletes had four or more years of training experience. The inclusion criteria were: adult subjects, tennis training experience of more than one year, subject‘s written informed consent. The exclusion criteria were as follows: previous injuries of the musculoskeletal system (within the preceding six months), the presence of other health problems and medical conditions which may have had an influence on the results of the tests used in this study (for example, a current pain complaint). All participants were informed about the procedures of this study and written informed consent was obtained from all subjects before participation.

Detailed data concerning the study population is presented in Table 1.

### 2.2. Methods

#### 2.2.1. Functional Movement Screen Test 

For the purpose of the functional assessment of the subjects, the Functional Movement Screen Test (FMS™) was used as well as core stability tests, which included the abdominal muscles endurance test, the lateral trunk muscles endurance test (the side bridge test), and the trunk extensors muscle endurance test (study 1). The FMS is a screening tool used to assess the functional fitness of athletes. The correct performance of the tests requires an appropriate level of stability and mobility, as well as coordination and balance [17,18]. 

The FMS test consists of seven movement tasks including: deep squat, hurdle step, in-line lunge, shoulder mobility, active straight-leg raise, trunk stability push-up, rotational stability. The FMS uses a simplified grading system. Each test is scored from 0 to 3 (with 3 points being the optimal score). Each test (movement pattern) has certain criteria that must be fulfilled in order to obtain the highest score. Three points are given if the individual can perform the movement without any compensations according to the test criteria. Two points are given if the individual can perform the movement but must use compensatory patterns. One point is given if the individual cannot perform the movement pattern even with compensations. Zero points are given if the individual has pain during any part of the movement [10,17]. The scores of each test are summed up for a composite score (with 21 points being the maximal score). According to the study of Cook et al. [17], low composite scores in this test, as well as side-to-side differences, are reported as increased injury risk factors. In some other authors‘ work it has been suggested that a composite score of 14 points (or lower) in this test indicated an increased risk of injury in healthy, active subjects [13,19,20]. There are also studies which have confirmed that the FMS test is a valid, sensitive, and reliable tool for the purpose of functional assessment in athletic populations [21,22,23]. 

#### 2.2.2. Core Stability Assessment

For the purpose of the assessment of core stability the lateral trunk muscles endurance test (the side bridge test), the abdominal muscles endurance test and the trunk extensors muscle endurance test were performed, as described by Donatelli and Carp [24].

1. In the lateral trunk muscles endurance test—the side bridge test (LTMET)—the subject is asked to assume the side bridge position during the test, with the body supported on the forearm and both feet. This test is used to assess of bilateral activity of hip and trunk stabilizers in the frontal plane (mainly isometric activity of the quadratus lumborum muscle) [24]
2. The abdominal muscles endurance test (ABSET) evaluates the strength of the rectus abdominis muscle. The examined person is asked to assume a sit-up position with the hands placed at the back of the neck and a trunk flexion of 60°. Then the subject is asked to hold the position. The norms described by Donatelli are 134 s for women and 136 s for men [24]
3. In the trunk extensors muscle endurance test (TEMET), the subject is asked to assume a prone position on the rehabilitation table with the body supported at the pelvis level and the arms crossed at the chest while the examiner stabilizes the pelvis and lower limbs. Then the subject is asked to extend the trunk and hold this position [24,25].

To collect the basic anthropometric and sociodemographic data, an original questionnaire with questions concerning age, gender, body weight, height, BMI (body weight [kg]/height [m^2^]) and training experience was used.

#### 2.2.3. Study Procedures

The study was conducted in 10 tennis clubs from Warsaw (Poland) and 4 clubs from Rzeszow (Poland). All the subjects participating in this study were amateur tennis players training and competing at a regional and national level. All the tests used in this study were performed twice by all subjects. The first evaluation (study 1) was part of the pre-season testing before the tennis outdoor season. The tests were performed in indoor tennis courts, at a temperature of about 21–22 °C, at the same time of day (from 4 to 8 PM). Two researchers (one experienced physiotherapist and one strength and conditioning coach), certified in the FMS method, were responsible for conducting all the tests. Athletes were asked to come for the examination on a predetermined day and time, wearing their regular sportswear. They were also asked not to perform any intense training or activity in the day before the assessment. The testing procedure was preceded by a short (10 min), moderate-intensity warm-up (including some running, dynamic stretching and agility drills). For the purpose of performing the FMS test the original FMS Test Kit was used (Functional Movement Systems Inc., Chatham, VA, USA). The core stability tests were performed by all the tennis players in random order, after completing the FMS test. All tests were presented and clearly explained to the subjects by the researchers. Besides this, each subject performed a testing trial of each test, for the purpose of familiarization. There were rest intervals of approximately five minutes between the core stability tests, as suggested by Nesser et al. [26]. 

After the first evaluation, the six-week core stability exercise program was introduced. The exercise program was mainly focused on the strengthening and stabilising of the lumbo-pelvic complex muscles and it consisted of: (1) isometric, voluntary activation of the trunk “deep stabilisers” in the supine position with the lower legs flexed at the hip and knees and with feet flat on the floor-active pelvis posterior tilt; (2) active straight leg-lowering exercise in supine position, with the focus on maintaining a neutral spine; (3) side plank (right and left side) with hip abduction; (4) isometric front plank; (5) deep squat; (6) “burpees”; and (7) “sumo squat” with hip abduction. The exercises were presented to the subjects with a special focus on proper technique. They also received a detailed exercise program, including a short warm-up routine, the number of sets and repetitions, and a detailed description of each exercise. All subjects in the study group were asked to perform the core stability exercise program every day for six weeks. All subjects participated in their regular tennis training (three times/week) througout the study. The second evaluation, including FMS and the core stability tests, using the same testing procedures, was performed after six weeks of the core stability training program (study 2).

#### 2.2.4. Statistical Analysis

All statistical analysis were performed using the Statistica 13.2. software (StatSoft Company, Kraków, Poland). Normal data distribution was verified using the Shapiro-Wilk test, and non-parametric versions of the statistical test were used. Basic descriptive statistics were used for the purpose of statistical analysis (mean value, standard deviation). For the purpose of comparing data between the first and second evaluations, the Wilcoxon signed-rank test was performed. For the comparison of two independent variables, the Mann–Whitney *U* test was used. Spearman’s rank correlation coefficient was used to assess correlation between the FMS test scores and the results of the core stability tests. The values of 0.70 and above were considered a strong correlation, 0.40–0.70 moderate, and below 0.40 a poor correlation [27]. The level of statistical significance was assumed at *p* < 0.05. All *p* values below 0.05 were considered a statistically significant (*p* < 0.05). An a priori power analysis revealed that a total sample size of 72–102 subjects would be needed to achieve a moderate effect size (0.5–0.6) and 80% power.

## 3. Results

Statistical analysis of the results in the first and second study (before and after introducing the core stability exercise program) revealed significant difference in FMS test results. The mean value of all the seven tests in FMS, as well as the FMS composite score, had significantly improved in both female and male tennis players (Table 2). The mean value in the FMS total score in female athletes in the first study was 14.58 ± 2.91, and in the second study 17.20 ± 1.68 (*p* < 0.001), while the scores were 14.44 ± 2.76 and 16.91 ± 1.36 (*p* < 0.001) in the male group. Detailed results are presented in Table 2.

Table 3 presents the results of the core stability tests before and after the core stability training program in the study group, sorted by the gender of the subjects. We observed a statistically significant increase in all test scores, in both women and men, although the average improvement after the six-week core stability exercise program was greater in male tennis players.

We observed a statistically significant improvement in all core stability tests results after the six-week training program in tennis players of both subgroups with regards to their training experience (1–3 years and 4 and more years) (Table 4).

We also observed similar results in the FMS composite scores before and after the six-week core stability training program with regards to tennis players’ training experience. In the group with one to three years of training experience, the initial FMS composite score was 12.55 ± 2.55, while in the second study it was 16.32 ± 1.25 (*p* < 0.001). In the the group with four or more years of training experience, the FMS composite score was 16.27 ± 1.67 in the first study, while it was 17.70 ± 1.45 (*p* < 0.001) in the second study after the six-week training program. In both groups the average improvement of the FMS composite score was statistically significant (Table 5).

Table 6 presents the differences in the FMS composite score and core stability test results, comparingtennis players with shorter and longer training experience in the first and second study. During the initial study the FMS composite score was significantly lower in the group of tennis players with shorter training experience compared to the group with four or more years of training experience (12.55 ± 2.55 and 16.27 ± 1.67, *p* < 0.001). There were no significant differences between these two groups in core stabilility test scores before the six week training program. However, statistical analysis revealed a significant differences in all test results (FMS composite score and core stability test results) after the training program. 

In the second study the average results of all tests improved in both groups of athletes, although by analysing the average improvement in the core stability tests results, we observed that improvement was greater in the group of athletes with longer training experience. This may suggest that they “responded“ better to the training program.

Table 7 provides data concerning correlations between the FMS composite score and each of the core stability tests during the second study, which was undertaken after the six-week training program. All the correlations were statistically significant, though moderate, with the correlation between the FMS composite score and the trunk extensors muscle endurance test results being the most significant of them all (R = 0.690).

## 4. Discussion

The FMS test and core stability tests are commonly used to assess an athlete‘s functional performance and injury risk [9,28]. In our research we used the FMS test and core stability tests to assess the effects of a six-week training program focused mainly on strengthening and stabilising of the lumbo-pelvic complex muscles in a group of athletes aged 18–23 (20.27 ± 1.68 years) practising tennis. The shortest training experience in our group ranged from one to three years (47.50% of the study group), with the rest of the group having four or more years of training experience (52.50% of the study group).

In numerous studies the researchers assessed the effects of various forms of training and prevention exercises on functional movement patterns and sports injury risk in many sports disciplines [29,30]. Šćepanović et al. [31] evaluated the effects of a six-week core strengthening exercise in a group of 36 female, untrained students, randomly assigned to two groups. In a specific, core strengthening exercise training group, they observed higher results in the FMS test, compared with a group of female students participating in a traditional exercise program.

Bonazza et al. [32] performed a scientific review of the literature concerning the effectiveness of FMS for assessing injury risk in athletes and individuals in military service. The authors suggested that FMS was a reliable, valid, and injury predictive tool, with lower scores on the test indicating increased injury risk in this group. Asgari et al. [33] presented a similar systematic review on the FMS test injury predictive value in the active, female groups. The authors confirmed the validity of the FMS test in scientific practice, although they emphasised that due to the insufficient number of studies among active females, there is still a need to perform such studies on a larger female, athletic population. In the authors’ own research, the results of statistical analysis revealed a significant improvement of all FMS test scores in the second study after a six-week core stability exercise program, both in male and female athletes.

Šiupšinskas et al. [34] assessed the association of pre-season musculoskeletal screening and functional test results (including FMS) with sports injuries in elite female basketball players. Researchers showed that impaired functional movement patterns (FMS test) as well as poor jump landing biomechanics (Landing Error Scoring System test-LESS) during pre-season screening tests were associated with an increased risk of lower limb injuries in female basketball players during the following season. The injured group had a lower composite FMS score (*p* = 0.0001) and a higher total LESS score (*p* = 0.028) than the non-injured group. However, the impairments of dynamic stability in the lower limbs (Y Balance test) were not associated with a higher injury rate in their study. The authors suggested that due to the fact that many different factors may contribute to increased injury risk in sport, a combination of the functional tests and performance-oriented tests should be used in pre-season injury risk assessment. They also indicated that systematic, specific functional training, including core stability exercises, may reduce the injury risk in female basketball players [34]. In our study we also observed a significant improvement in core stability tests results after the six-week training program, which may have a positive impact on reducing injury risk.

Xio et al. [35] presented results of the systematic review concerning the effects of exercise on physical fitness characteristics in young tennis players. The results of their review indicated that specific exercise training could significantly increase the physical fitness of young tennis players in terms of speed and agility, although there was a lack of evidence or conflicting evidence about strength and flexibility, as well as power, in this group. The results of our study indicated that a specific, functional exercise program could improve functional movement patterns (as assessed with the FMS test) and core stability test results. The FMS composite score and core stability test results were significantly higher after the six-week training program in our study group.

Fernandez et al. [36] have assessed the effects of a five-week training program including specific neuromuscular exercises on physical performance in a group of young, elite tennis players. The study group consisted of 18 young, male tennis players (mean age 15.09 ± 1.16 years). They were randomly assigned to two groups. The first group performed specific, neuromuscular training as a part of their warm-up before tennis training sessions, while the other group performed the same training program after their tennis training sessions. The inclusion of neuromuscular training before regular tennis training had a positive effect on most physical fittnes measures in the second study, after completing the training program (i.e., jump, sprint, change of direction-agility, as well as upper-body power). Interestingly, performing the same exercise after the regular tennis training was not associated with the same improvement in physical performance test results. The authors also emphasised the importance of specific core stability training as well as trunk and shoulder exercises in combination with neuromuscular training as a part of a regular warm-up routine, in order to improve tennis-related physical performance variables. These results were similar to our study, where we observed significant improvement in functional movement patterns and core stability test results, after the six-week core stability exercise program.

Rey et al. [37] presented slightly different conclusions in their research. The aim of their study was to assess the effect of the structured warm-up program (FIFA 11+), compared to a standard warm-up. on fundamental movement patterns in the FMS test in a group of amateur, male footballers. The 11+ warm-up routine in the intervention group lasted for around 25 min and it was performed three times a week for a six week period, instead of the regular, basic warm-up in the control group. The FMS total score improved significantly in both the intervention and control group, although there were no significant differences in overall performance beetwen the two groups. These results may suggest that specific and structured warm-up programs (i.e., 11+) may not result in additional, significant improvements in fundamental movement patterns, other than those achieved using a standard, sport discipline specific warm-up in soccer players.

Hoover et al. [38] performed research concerning the injury predictive validity of the FMS test in a group of 32 male and female professional basketball players. The aim of the study was to assess the risk of non-contact injuries in professional basketball players based on FMS scores. The basketball players were initially assessed using FMS during a pre-season training camp. Each athlete was then monitored throughout the season with number and type of injury and time lost due to injury recorded. The authors of the study used linear regression models to analyse predictive ability of the FMS score. In all models the total FMS score did not turn out to be a significant injury predictor (*p* > 0.05). In contrast to much other research, the results of their study did not confirm FMS test injury predictive validity in this sample of professional basketball players.

Two Polish authors, Koźlenia and Domaradzki [39], evaluated in their study the association between physical performance test results and the quality of fundamental movement patterns assessed with the FMS test among young female Physical Education students. The authors suggested that there were still some conflicting results concerning the association between physical performance and quality of movement. However, both these factors could affect injury risk. In their study physical performance tests evaluated strength, power and flexibility. The main findings indicated that flexibility had the strongest and most statistically significant impact on the FMS composite score (ß = 0.25, *p* = 0.0106) and asymmetries in the test (ß = −0.30, *p* = 0.0014). Additionally, a significant, negative correlation of abdominal muscle strength and FMS asymmetries were observed (ß = −0.29, *p* = 0.0027).

There are some other studies which use FMS to evaluate the effectiveness of different training and intervention programs in athletic populations. [14,15,16]. Our study results have also revealed a significant improvement in FMS results following the six-week training program in a group of tennis players with different training experience (one to three years as well as four or more years of training experience). The recent study of Fernandez et al. [40] assessed the effect of different types of warm-up exercises (neuromuscular vs. dynamic warm-up) on physical performance measures in young tennis players. The results showed that both warm-up routines significantly improved some of the physical performance test scores in the both study groups (sprinting performance, bilateral and unilateral counter movement jump, overhead medicine ball throw and some shoulder strength and ROM values). However, the neuromuscular warm-up produced greater gains in most of the performance-oriented test results (i.e., 5–10 m sprint, counter movement jump, overhead medicine ball throw and serve speed). The results of their study may indicate that a systematic, neuromuscular warm-up routine (including mobility, core stability, and shoulder strength exercises, combined with neuromuscular-related exercises, e.g., plyometric, acceleration/deceleration, and change of direction agility drills) can be recommended in young tennis players [40].

The results of our study showed positive effects of the six-week core stability training program in a homogeneous group of adult tennis players. We observed significant improvement in functional movement patterns measured with the FMS test, as well as improved core stability (trunk muscle endurance test results), both in male and female athletes. According to some other studies results, this may have a positive impact on tennis-related physical performance and reduced injury risk. However, due to the lack of strong evidence and some conflicting results, the relationship between the quality of movement patterns, core stability and injury risk as well as tennis-specific physical performance requires further research in this study group.

There are some limitations in our study that need to be addressed in the future research. The major limitations of our study concern the lack of a control group that had not participated in the six-week core stability exercise program. Moreover, our study was performed in a particular group of amateur, adult tennis players. Therefore future studies should include different age groups (including youth tennis players) and ability level.

## 5. Conclusions

The results of our study revealed a positive impact of a six-week core stability exercise program on the quality of fundamental movement patterns (as meassured by the FMS test) as well as core stability test results in a group of adult male and female tennis players. Taking into account the findings of our research and the results of studies by other authors, core stability exercises can be recomended in this group of tennis players as a valuable and effective supplement to regular tennis training.

## Figures and Tables

**Table 1:** Characteristics of the study population.

	Women (*n* = 74)	Men (*n* = 86)	All (*n* = 160)
Age (±; SD)	20.29 ± 1.83	20.26 ± 1.55	20.27 ± 1.68
Body weight (±; SD)	54.80 ± 4.41	62.69 ± 7.14	59.04 ± 7.19
Height (±; SD)	164.86 ± 3.77	171.75 ± 6.04	168.57 ± 6.16
BMI (*n*; %)			
Normal	74; 100.00	86; 100.00	160; 100.00
Overweight	0; 00.00	0; 00.00	0; 00.00
Obesity	0; 00.00	0; 00.00	0; 00.00
Training experience (*n*; %)			
1–3 years	35; 47.29	41; 47.67	76; 47.50
4 and more years	39; 52.71	45; 52.33	84; 52.50

*n*-sample size; ±;SD–mean value and standard deviation,; BMI–Body Mass Index.

**Table 2:** The FMS test results before and after core stability training program.

	Before (Study 1)										After (Study 2)										
	Women (*n* = 74)					Men (*n* = 86)					Women (*n* = 74)					Men (*n* = 86)					All (*n* = 160)
	III	II	I	0	x~	III	II	I	0	x~	III	II	I	0	x~	III	II	I	0	x~	*p* *
DS (*n*)	3	28	43	-	1.46	0	36	50	-	1.42	29	40	5	-	2.32	37	33	16	-	2.24	*p* < 0.001
HS (*n*)										-											
L	32	30	12	-	2.27	28	47	11	-	2.20	43	25	6	-	2.50	44	38	4	-	2.47	*p* < 0.001
R InLL (*n*)	35	35	4	-	2.42	29	51	6	-	2.27	48	26	-	-	2.65	41	44	1	-	2.47	*p* < 0.001
L	36	31	7	-	2.39	31	45	10	-	2.24	37	35	2	-	2.47	38	47	1	-	2.43	*p* < 0.001
R	33	37	4	-	2.39	30	56	-	-	2.35	35	37	2	-	2.45	33	53		-	2.38	*p* < 0.001
SM (*n*)																					
L	48	19	7	-	2.55	51	25	10	-	2.48	54	19	1	-	2.72	52	31	3	-	2.57	*p* < 0.001
R	38	27	9	-	2.39	47	29	10	-	2.43	49	22	3	-	2.65	50	32	4	-	2.53	*p* < 0.001
ASLR (*n*)																					
L	31	31	11	1	2.24	45	29	6	6	2.31	31	40	3	-	2.41	46	35	5	-	2.48	*p* < 0.001
R	15	28	30	1	1.77	32	29	12	3	2.18	30	35	9	-	2.32	35	44	7	-	2.33	*p* < 0.001
TSPU (*n*)	34	25	7	8	2.15	25	40	11	10	1.93	34	28	9	3	2.34	30	45	11	-	2.22	*p* < 0.001
RS (*n*)																					
L	31	43	-	-	2.42	46	39	1	-	2.52	39	35	-	-	2.59	60	26	-	-	2.70	*p* < 0.001
R	31	43	-	-	2.42	36	50	-	-	2.42	52	22	-	-	2.73	48	38	-	-	2.44	*p* < 0.001
TOTAL (±; SD)	14.58 ± 2.91					14.44 ± 2.76					17.20 ± 1.68					16.91 ± 1.36					*p* < 0.001

DS-Deep Squat; HS-Hurdle Step; InLL-In Line-Lunge; SM-Shoulder Mobility; ASLR-Active Straight Leg Raise; TSPU-Trunk Stability Push Up; RS-Rotational Stability; L-left side; R-right side; TOTAL–FMS composite score test; *n*–sample size; **x~-** mean value; III-3 points in FMS test (maximal value); II-2 points in FMS test; I-1 point in FMS test; 0-zero points in FMS test; *p* *-Wilcoxon signed-rank test result.

**Table 3:** Results of the core stability tests before and after core stability training program.

	Before (Study 1)		After (Study 2)		*p* *
	Women (*n* = 74)	Men (*n* = 86)	Women (*n* = 74)	Men (*n* = 86)	All (*n* = 160)
LTMET (±; SD)	69.14 ± 7.69	82.83 ± 8.10	77.27 ± 8.92	95.69 ± 9.25	*p* < 0.001
ABSET (±; SD)	127.53 ± 6.46	128.23 ± 8.19	137.41 ± 9.22	143.37 ± 12.35	*p* < 0.001
TEMET (±; SD)	171.929 ± 11.27	138.72 ± 14.26	185.5 ± 12.85	160.02 ± 19.58	*p* < 0.001

LTMET-lateral trunk muscles endurance test; ABSET–abdominal muscles endurance test; TEMET–trunk extensors muscle endurance test; *n*–sample size; ±; SD–mean value and standard deviation of the core stability tests results (in seconds); *p* *-Wilcoxon signed-rank test result.

**Table 4:** Results of the core stability tests before and after core stability training, sorted by training experience.

	Training Experience					
	1–3 Years (*n* = 76)			4 and More Years (*n* = 84)		
	Before (Study 1)	After (Study 2)	*p* *	Before (Study 1)	After (Study 2)	*p* *
LTMET (±; SD)	75.61 ± 10.63	82.96 ± 13.46	*p* < 0.001	77.29 ± 10.27	90.98 ± 11.21	*p* < 0.001
ABSET (±; SD)	127.69 ± 7.34	138.14 ± 13.81	*p* < 0.001	128.09 ± 7.55	142.84 ± 8.06	*p* < 0.001
TEMET (±; SD)	152.63 ± 20.36	166.53 ± 22.25	*p* < 0.001	155.38 ± 21.67	176.57 ± 18.78	*p* < 0.001

LTMET-lateral trunk muscles endurance test; ABSET–abdominal muscles endurance test; TEMET–trunk extensors muscle endurance test; *n*–sample size; ±; SD–mean value and standard deviation of the core stability tests results (in seconds); *p* *-Wilcoxon signed-rank test result.

**Table 5:** FMS composite score before and after core stability training sorted by training experience.

	Training Experience					
	1–3 Years (*n* = 76)			4 and More Years (*n* = 84)		
	Before (Study 1)	After (Study 2)	*p* *	Before (Study 1)	After (Study 2)	*p* *
TOTAL FMS (±; SD)	12.55 ± 2.55	16.32 ± 1.25	*p* < 0.001	16.27 ± 1.67	17.70 ± 1.45	*p* < 0.001

TOTAL FMS–FMS composite score; *n*–sample size; ±; SD–mean value and standard deviation; *p* *-Wilcoxon signed-rank test result.

**Table 6:** The FMS composite score and core stability test results before and after the training program in groups of athletes with shorter and longer training experience.

	Training Experience		
	1–3 Years (*n* = 76)	4 and More Years (*n* = 84)	*p* *
			All (*n* = 160)
**Before (study 1)**			
TOTAL FMS (±; SD)	12.55 ± 2.55	16.27 ± 1.67	*p* < 0.001
LTMET (±; SD)	75.61 ± 10.63	77.29 ± 10.27	*p* < 0.188
ABSET (±; SD)	127.69 ± 7.34	128.09 ± 7.55	*p* < 0.830
TEMET (±; SD)	152.63 ± 20.36	155.38 ± 21.67	*p* < 0.414
**After (study2)**			
TOTAL FMS (±; SD)	16.32 ± 1.25	17.70 ± 1.45	*p* < 0.001
LTMET (±; SD)	82.96 ± 13.46	90.98 ± 11.21	*p* < 0.001
ABSET (±; SD)	138.14 ± 13.81	142.84 ± 8.06	*p* < 0.001
TEMET (±; SD)	166.53 ± 22.25	176.57 ± 18.78	*p* < 0.001

TOTAL FMS–FMS composite score; LTMET-lateral trunk muscles endurance test; ABSET–abdominal muscles endurance test; TEMET–trunk extensors muscle endurance test; *n*–sample size; ±; SD–mean value and standard deviation; *p* *-Mann–Whitney *U* test.

**Table 7:** Correlations between the FMS composite score and core stability test scores.

	R-Spearman	*p* *
TOTAL FMS & LTMET	0.450	*p* < 0.001
TOTAL FMS & ABSET	0.450	*p* < 0.001
TOTAL FMS & TEMET	0.690	*p* < 0.001

TOTAL FMS–FMS composite score; LTMET-lateral trunk muscles endurance test; ABSET–abdominal muscles endurance test; TEMET–trunk extensors muscle endurance test; R-Spearman’s rank correlation coefficient value. *p* *-the result of *t* test-test of significance for Spearman’s rank correlation coefficient.

## Data Availability

Not applicable.
